# Temperature-Dependent Growth Characteristics of Nb- and CoFe-Based Nanostructures by Direct-Write Using Focused Electron Beam-Induced Deposition

**DOI:** 10.3390/mi11010028

**Published:** 2019-12-25

**Authors:** Michael Huth, Fabrizio Porrati, Peter Gruszka, Sven Barth

**Affiliations:** Institute of Physics, Goethe University, 60438 Frankfurt am Main, Germany; porrati@physik.uni-frankfurt.de (F.P.); gruszka@physik.uni-frankfurt.de (P.G.); barth@physik.uni-frankfurt.de (S.B.)

**Keywords:** focused electron beam induced deposition, precursor residence time, continuum model

## Abstract

Focused electron and ion beam-induced deposition (FEBID/FIBID) are direct-write techniques with particular advantages in three-dimensional (3D) fabrication of ferromagnetic or superconducting nanostructures. Recently, two novel precursors, HCo${}_{3}$Fe(CO)${}_{12}$ and Nb(NMe${}_{3}$)${}_{2}$(N-*t*-Bu), were introduced, resulting in fully metallic CoFe ferromagnetic alloys by FEBID and superconducting NbC by FIBID, respectively. In order to properly define the writing strategy for the fabrication of 3D structures using these precursors, their temperature-dependent average residence time on the substrate and growing deposit needs to be known. This is a prerequisite for employing the simulation-guided 3D computer aided design (CAD) approach to FEBID/FIBID, which was introduced recently. We fabricated a series of rectangular-shaped deposits by FEBID at different substrate temperatures between 5 ${}^{{}^{\circ}}$C and 24 ${}^{{}^{\circ}}$C using the precursors and extracted the activation energy for precursor desorption and the pre-exponential factor from the measured heights of the deposits using the continuum growth model of FEBID based on the reaction-diffusion equation for the adsorbed precursor.

## 1. Introduction

Direct-write nano-fabrication by focused electron and ion beam-induced deposition (FEBID/FIBID) has become one of the most promising approaches for the realization of two- and three-dimensional (3D) functional structures with particular relevance for the fields of nano-magnetism [1,2,3,4], nano-optics [5,6], and superconductivity of nanostructures [7,8]. This is due to essentially two important advantages that FEBID/FIBID have as compared to other technologies [9], which are their applicability on virtually any surface and the flexibility to fabricate wireframe- [3] as well as sheet-like [10] structures with sub-100 nm resolution. In order to fully develop the potential of FEBID/FIBID for the mentioned research areas, a reliable transfer of desired 3D structures into real 3D nano-objects is mandatory. Over the last few years, this has led to the evolution of a simulation-guided 3D computer aided design (CAD) approach which was pioneered by Fowlkes and collaborators [11] and has been further developed towards a reliable instrument for 3D nano-fabrication even of complex nano-architectures, as was recently reviewed by Winkler et al. [12]. For the fields of 3D nano-magnetism and nano-superconductivity, the precursors Co${}_{2}$(CO)${}_{8}$ [1], Fe${}_{2}$(CO)${}_{9}$ [13], HCo${}_{3}$Fe(CO)${}_{12}$ [3] (FEBID), W(CO)${}_{6}$ [14], and Nb(NMe${}_{3}$)${}_{2}$(N-*t*-Bu) [7] (FIBID) have been used and proven to be particularly suited. With a view to the simulation-guided 3D CAD approach to nano-fabrication which uses the continuum model of FEBID/FIBID growth, a set of simulation parameters is required; see Reference [15] for a recent review. Here, we focus on the precursors HCo${}_{3}$Fe(CO)${}_{12}$ and Nb(NMe${}_{3}$)${}_{2}$(N-*t*-Bu), recently introduced by us, and provide the activation energies and pre-exponential factors for thermally induced precursor desorption from a series of FEBID experiments performed under low beam current conditions (I=30 pA) on Si(100)/SiO${}_{2}$ substrates held at different temperatures between 5 ${}^{{}^{\circ}}$C and 24 ${}^{{}^{\circ}}$C. By comparison with deposition results obtained under high beam current conditions (I=1.6 nA), we highlight the morphological consequences imposed by the change of the growth regime from reaction-rate limited or diffusion enhanced to mass-transport limited. We note that similar experiments with FIBID, as would seem to be natural for the Nb-precursor, cannot be done for deduction of the activation energies due to the unavoidable etching effect in parallel to the growth; see Reference [15] for details.

## 2. Results

### 2.1. Fabrication, Height, and Composition Analysis

**Figure 1:**
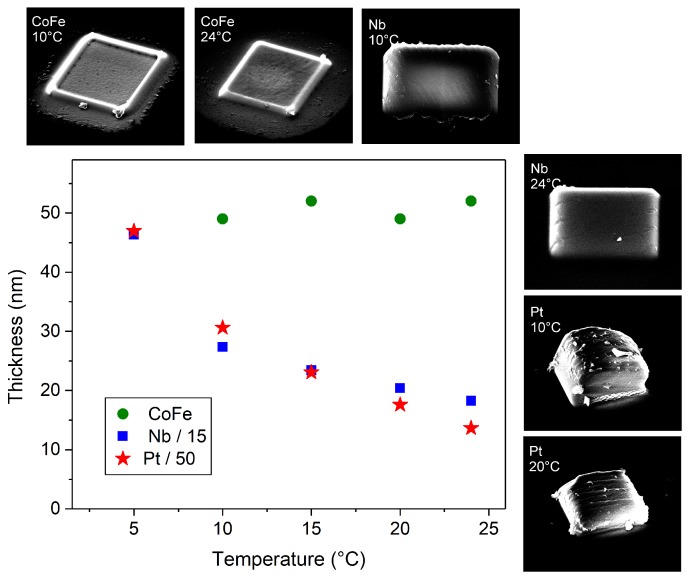
Thickness vs. temperature of samples grown at 5 keV and 1.6 nA beam current (experiment *a*). Green points: samples fabricated with the CoFe-precursor. Blue squares: samples grown using the Nb-precursor. Red stars: deposits grown with the Pt-precursor. Distributed about the main panel, a selection of scanning electron microscope (SEM) images is shown for samples fabricated at the substrate temperatures as indicated.

The fabrication of the samples was carried out using 5 keV for the electron beam energy and either 1.6 nA (experiment *a*) or 30 pA (experiment *b*) for the electron beam current at different substrate temperatures between 5 ${}^{{}^{\circ}}$C and 24 ${}^{{}^{\circ}}$C; see section Materials and Methods for details. In Figure 1, we report results of experiment *a*. In particular, we depict the thickness versus the substrate temperature for deposits written with the CoFe-, Nb-, and Pt- precursors. The samples written with HCo${}_{3}$Fe(CO)${}_{12}$ have a thickness of about 50 nm, measured as average values taken at the center of the deposits by atomic force microscopy. The height does not depend on the substrate temperature. The deposits written with Nb(NMe${}_{2}$)${}_{3}$(N-*t*-Bu) and Me${}_{3}$CpMePt show similar temperature-dependent behaviors with thickness increasing as the temperature is lowered. Note that the condensation temperatures of HCo${}_{3}$Fe(CO)${}_{12}$ and Nb(NMe${}_{2}$)${}_{3}$(N-*t*-Bu) (or Me${}_{3}$CpMePt) are about 10 ${}^{{}^{\circ}}$C and 5 ${}^{{}^{\circ}}$C, respectively. Therefore, depositions at lower temperatures were not carried out.

The elemental composition was investigated by energy dispersive X-ray spectroscopy (EDX) on thicker reference samples in order to avoid excitation of the substrate. The composition of the deposits prepared at 24 ${}^{{}^{\circ}}$C using HCo${}_{3}$Fe(CO)${}_{12}$ was about Co 65 at%, Fe 15 at%, C 12 at%, and O 8 at%, in accord with the values reported by us previously for the beam parameters used here [16]. No temperature dependence of the composition was detected within the experimental error of the EDX measurements, which we estimate to be about 2 at%. The composition of the samples written using Nb(NMe${}_{2}$)${}_{3}$(N-*t*-Bu) was about Nb 11 at%, C 65 at%, and N 24 at%, very similar to the values reported by us recently [7]. Finally, for the samples fabricated using the Pt-precursor, we found the composition Pt 17 at% and C 83 at%, as known from several investigations; see, e.g., Reference [17]. For the samples prepared with the Nb- and Pt-precursor, we measured an increase of the carbon content of about 3 at% as the substrate temperature during growth was reduced from 24 ${}^{{}^{\circ}}$C to 5 ${}^{{}^{\circ}}$C.

**Figure 2:**
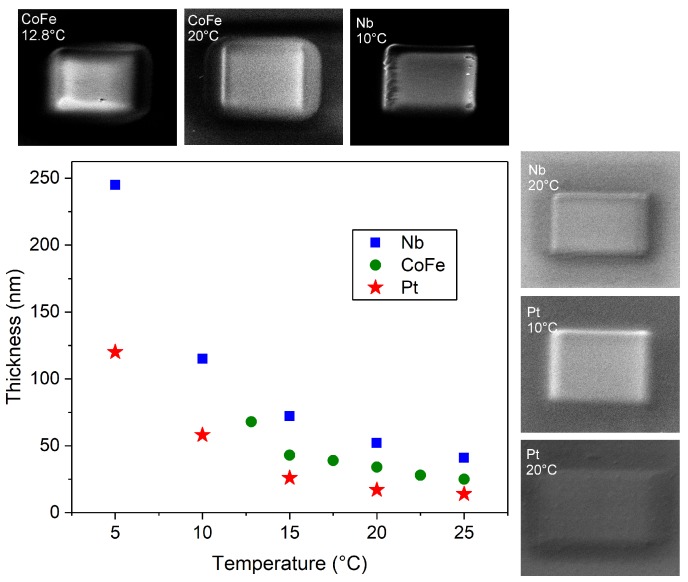
Thickness vs. temperature of samples grown at 5 keV and 30 pA beam current (experiment *b*). Green points, blue squares, and red stars refer to samples grown using the CoFe-, Nb-, and Pt-precursor, respectively. Distributed about the main panel, a selection of SEM images is shown for samples fabricated at the substrate temperatures as indicated.

In Figure 2, we report the results of experiment *b*. In contradistinction to what was observed at higher beam current, now the deposit thickness increases as the substrate temperature is reduced for all three precursors. This thickness increase is analyzed in more detail in the following subsection. Note that, for HCo${}_{3}$Fe(CO)${}_{2}$ and Nb(NMe${}_{2}$)${}_{3}$(N-*t*-Bu) the data point at the lowest substrate temperature, respectively, fall out-of-order (see next subsection), which we attribute to the partial condensation of the precursors. Due to the low electron current used in experiment *b*, sample thicknesses are smaller than those found in experiment *a*. Therefore, we did not perform EDX measurements as the deposition time to reach sufficient deposit heights, in particular at higher substrate temperature, would have been very long and the deposit composition is not the main focus of this work.

**Figure 3:**
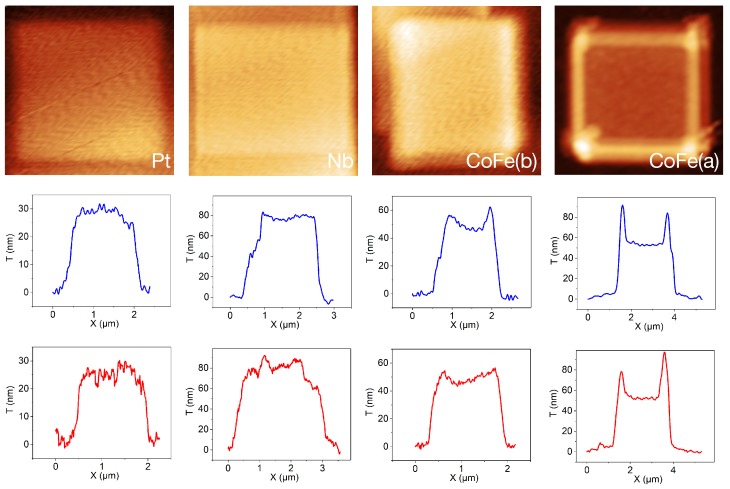
Topography (upper row) and line scans (middle and lower row) from atomic force microscope (AFM) measurements carried out on samples prepared at 15 ${}^{{}^{\circ}}$C using the precursors as indicated: For each sample, a scan in the vertical direction (in blue) and horizontal direction (in red) is shown. The last two column of pictures, labeled *CoFe (b)* and *CoFe (a)*, refer to samples prepared in experiments *b* and *a*, respectively.

In Figure 3, we show a selection of atomic force microscope (AFM) measurements. All topography images but the last shown in the first row refer to experiment *b*. The last image refers to experiment *a*. For all deposits, the substrate temperature was set to 15 ${}^{{}^{\circ}}$C. For each sample, we also depict two line-scans in the second and third rows, taken vertically (blue line) and horizontally (red line), respectively. The samples grown with the Pt- and Nb-precursor show a uniform height, which is characteristic of the reaction-rate-limited growth regime, see, e.g., Reference [18]. In contradistinction, the samples grown with the CoFe-precursor show a nonuniform height distribution. This is most pronounced at the the higher beam current and indicates the crossover from the diffusion-enhanced to the mass-transport-limited regime. As a consequence, lateral precursor transport by diffusion from regions out of the beam patterning region causes a higher growth rate at the edges of the deposit; see, e.g., Reference [15].

### 2.2. Analysis of Thermally-Induced Desorption

Within the continuum model of FEBID/FIBID growth, the time and spatial dependence of the precursor coverage is governed by the following reaction-diffusion equation [15]

(1) $$\frac{\partial \theta }{\partial t}=D\left(\frac{\partial^{2}\theta }{\partial x^{2}}+\frac{\partial^{2}\theta }{\partial y^{2}}\right)-\frac{\theta }{\tau }-\Phi_{e}\sigma \theta +s\frac{\Phi_{g}}{n_{0}}\left(1-\theta \right)\;,$$

where θ represents the precursor density in units $n_{0}$; the maximum precursor surface density, *D*, is the temperature-dependent diffusion coefficient; τ is the temperature-dependent precursor residence time; $\Phi_{e}$ is the electron flux density; σ is the averaged dissociation cross section; *s* is the precursor sticking coefficient; and $\Phi_{g}$ is the precursor flux density. We assume that only a maximum of one monolayer can adsorb, which is generally a valid assumption for FEBID precursors [15]. In the present case of flat deposits, the spatial derivatives can be taken for a flat surface that does not evolve in time, which facilitates the numerical solution of this partial differential equation.

We aim to extract the parameters $E_{A}$ and $\kappa_{0}$ governing the temperature-dependent residence time τ via a thermally activated behavior according to

(2) $$1/\tau =\kappa_{0}\exp\left(-E_{A}/k_{B}T\right)\;.$$

This is most easily accomplished in the case of a stationary equilibrium coverage $\theta_{0}$ obtained from Equation (1) under beam-off condition without the diffusion term, which is not appreciably reduced over one dwell event of duration $t_{D}$. Solving for $\theta_{0}$ from Equation (1) in this stationary state, we obtain

(3) $$\theta_{0}=s\frac{\Phi_{g}}{n_{0}}\frac{1}{1/\tau +s\Phi_{g}/n_{0}}\;.$$

For $\theta \approx \theta_{0}$, corresponding to the reaction-rate limited growth regime, the local height *h* of the deposit is given by

(4) $$h=\theta_{0}n_{0}\times \Phi_{e}\sigma VNt_{D}\;,$$

where *N* denotes the number of repetitions of the dwell event at the given position and *V* is the volume of deposit per dissociated precursor molecule. Using Equation (3) and solving for 1/τ, one obtains

(5) $$\frac{1}{\tau }=\kappa_{0}\exp\left(-E_{A}/k_{B}T\right)=s\Phi_{g}\left(\frac{\Phi_{e}\sigma VNt_{D}}{h}-\frac{1}{n_{0}}\right)\equiv F\left(h\right)\;,$$

which is the basis for our analysis. We now use the measured deposit heights (center region) at the different substrate temperatures and take the precursor-specific parameters listed in Table 1. For the beam, we assume a Gaussian shape with full-width at half maximum of 7 nm corresponding to a well-focused beam at 5 kV and 30 pA beam current (experiment *b*), taking the secondary electron (SE-I) exit region of the primary beam with 2 nm beam diameter into account. This leads to an electron flux area density of $3.4\times 10^{6}\;$ nm${}^{-1}$s${}^{-1}$. The precursor flux we calculate from the chamber pressure increases (see Methods and Materials section) following Reference [19]. For the sticking coefficient, which can be assumed to be temperature independent [20], we take s=1, but refer to the Discussion section regarding this assumption. The dwell time is $t_{D}=$ 1 μs, and the loop number *N* is $6\times 10^{4}$ for the CoFe-and Nb-precursor and $3\times 10^{4}$ for the Pt-precursor, respectively.

**Figure 4:**
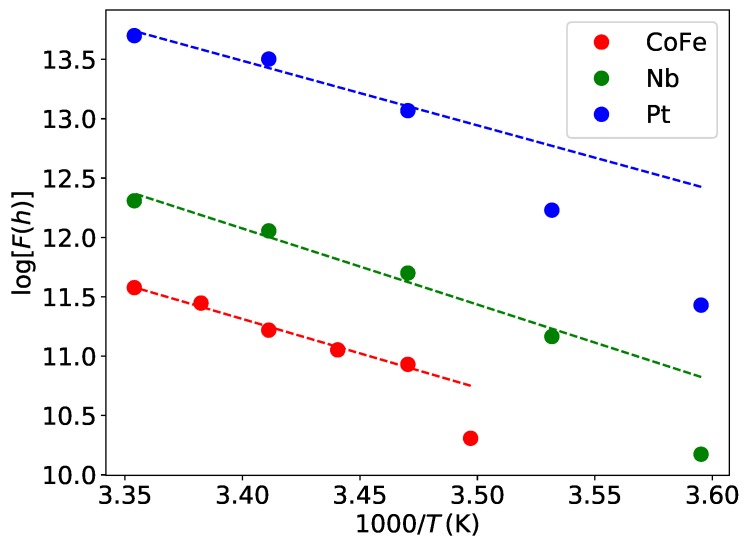
Logarithm of F(h), as defined in Equation (5), plotted vs. 1/T for the CoFe-, Nb-, and Pt-precursor as indicated. The dashed lines represent linear fits of the data excluding the lowest temperature data point for the CoFe and Nb deposits and the two low-temperature data points for Pt deposits.

In Figure 4, the quantity F(h), as defined in Equation ( 5), is plotted vs. the substrate temperature in Arrhenius representation for the three precursors. Evidently, for the CoFe-and Nb-precursor, a linear dependence is quite apparent if the data point taken at the lowest substrate temperature is excluded, as the CoFe- and Nb-precursors show deviating growth behavior due to beginning condensation at the lowest substrate temperature. For the Pt-precursor, we only use the three data points taken at the highest substrate temperature and note that the focus of this work is not on the Pt-precursor for which a careful analysis for the activation energy and pre-exponential factor has been previously performed [20].

From linear fits of the data, we extract the activation energies and pre-exponential factors as listed in Table 2.

## 3. Discussion

Thermal desorption analysis for FEBID/FIBID precursors relies on the knowledge of several precursor-specific parameters as listed in Table 1 in the present case. In particular, two parameters are typically only approximately known, namely the dissociation cross section σ and the sticking coefficient *s*. With regard to σ, gas phase dissociation studies as well as surface science studies can provide reasonable estimates, and these were used here for the Pt- and CoFe-precursors [22,23]. The sticking coefficient enters Equation (5) as a prefactor in F(h), so that the deduced $\kappa_{0}$ parameter scales directly with *s*, whereas the deduced activation energy $E_{A}$ does not depend on *s*. Once $E_{A}$ is determined by the analysis above, *s* can therefore be recalibrated by comparing numerical solutions of Equation (1) to the measured height in the limit of negligible diffusion. However, considering the line profile shapes of the CoFe deposits as the substrate temperature is reduced (see Figure 3), it is quite apparent that growth moves into the diffusion-enhanced regime as the substrate temperature is lowered. The height data points which can therefore be used for recalibrating $\kappa_{0}$ by choosing a different sticking coefficient can only approximately provide an estimate of *s*.

Recent work by Cullen and collaborators [20,24] has shown how both the activation energies and pre-exponential factors for desorption and diffusion can be deduced from FEBID experiments under stationary beam conditions if certain prerequisites are fulfilled. This approach may also be applied to the CoFe- and Nb-precursor to deduce the temperature dependence of *D*. This will then allow to take properly into account how beam-induced heating might change the growth rates for 3D growth using the CoFe- and Nb-precursor due to increased desorption and faster diffusion, as was previously investigated for the Pt-precursor [25].

## 4. Materials and Methods

### 4.1. Precursors

For our experiments, we employed the precursors HCo${}_{3}$Fe(CO)${}_{12}$, Nb(NMe${}_{2}$)${}_{3}$(N-*t*-Bu), and Me${}_{3}$CpMePt. The solid HCo${}_{3}$Fe(CO)${}_{12}$ precursor was synthesized in a slightly modified procedure [16] to the originally described methodology by Chini et al. [26] and finally recrystallized from toluene. The colorless Nb-precursor was prepared in a modified procedure [7] to the published salt elimination reaction described by Baunemann et al. [27]. Purification can be carried out by sublimation at reduced pressure ($∼10^{-3}$ mbar) or by distillation ($∼10^{-1}$ mbar) and temperatures between 50 and 76 ${}^{{}^{\circ}}$C. The Pt-precursor was purchased from Sigma-Aldrich and used as supplied.

### 4.2. Fabrication and Height and Composition Analysis

The samples were fabricated by FEBID in a dual beam SEM/FIB (FEI, Nova Nanolab 600, Hillsboro, OH, USA) equipped with a Schottky electron emitter. The precursors were injected in the SEM via capillaries with 0.5 mm inner diameters in close proximity to the focus of the electron beam on the substrate surface. The distance between the capillary exit and the substrate surface was about 100 μm. The samples were grown on Si (100) (p-doped)/SiO${}_{2}$ (200 nm) substrates. The precursors HCo${}_{3}$Fe(CO)${}_{12}$, Nb(NMe${}_{2}$)${}_{3}$(N-*t*-Bu), and Me${}_{3}$CpMePt were heated to 64 ${}^{{}^{\circ}}$C, 35 ${}^{{}^{\circ}}$C, and 44 ${}^{{}^{\circ}}$C, respectively. The base pressure of the SEM was 5 × 10${}^{-7}$ mbar. The depositions took place at 6 × 10${}^{-7}$ mbar, 8 × 10${}^{-7}$ mbar, and 1 × 10${}^{-6}$ mbar, respectively, for the three precursors. The planar sizes of the nanostructures were 2 μm× 2 μm and 1.5 μm× 1.5 μm for experiments *a* and *b*, respectively. The patterning strategy was serpentine, and the patches were always patterned from a single direction. The thickness of the nanostructures was controlled by setting the loop number *N*. The electron beam parameters for experiment *a* were 5 keV, 1.6 nA, 1 μs, and 20 nm for beam energy, beam current, dwell time, and pitch, respectively. *N* was $2\times 10^{4}$ for all samples. For experiment *b*, we used 5 keV, 30 pA, 1 μs, and 20 nm, respectively. *N* was $6\times 10^{4}$ for the samples grown using the CoFe- and Nb-precursor and $N=3\times 10^{4}$ for the Pt-precursor. The depositions were carried out at different substrate temperatures between 5 ${}^{{}^{\circ}}$C and 24 ${}^{{}^{\circ}}$C, employing a self-made cryo-stage made from copper. The Cu stage was cooled using a strand of Cu wires connected to a cold finger held at N${}_{2}$ lq. temperature. A heating foil placed under the Cu stage allowed to stabilize the required temperature, which was measured by a Pt-1000 sensor located in close proximity to the sample. All depositions were carried out by first cooling the cryo-stage to about 5 ${}^{{}^{\circ}}$C and by then heating it up to the target temperature. The deposit thicknesses were determined by ex situ atomic force microscope (AFM) (Nanosurf, easyscan2, Liestal, Switzerland) in dynamic mode. The composition of the samples was investigated by energy dispersive X-ray spectroscopy (EDX). 

## Figures and Tables

**Table 1:** Table of precursor-specific parameters used to deduce $E_{A}$ and $\kappa_{0}$ by employing Equation (5): *d* is the average precursor molecule diameter from which $n_{0}$ is calculated as $n_{0}=1/\pi {\left(d/2\right)}^{2}$. The volume of the deposit per dissociated precursor molecule *V* was calculated from average densities and molar masses using the deposit compositions as measured in independent X-ray spectroscopy (EDX) experiments. The cross sections are taken from References [21,22] for the Pt- and CoFe-precursor, respectively. For the Nb-precursor, the cross section is not known and was estimated, as typical cross sections are between 0.01 and 0.02 nm${}^{2}$ for focused electron beam-induced deposition (FEBID) precursors [22].

Precursor	*d* (nm)	$n_{0}$ (nm${}^{-2}$)	$\Phi_{g}$ (nm${}^{-2}$s${}^{-1}$)	σ (nm${}^{2}$)	*V* (nm${}^{3}$)
HCo${}_{3}$Fe(CO)${}_{12}$	0.83	1.85	$7.3\times 10^{2}$	0.011	0.05
Nb(NMe${}_{3}$)${}_{2}$(N-*t*-Bu)	0.77	2.15	$1.7\times 10^{3}$	0.015	0.13
Me${}_{3}$CpMePt	0.78	2.09	$2.7\times 10^{3}$	0.022	0.2

**Table 2:** Table of activation energies and pre-exponential factors for the CoFe-, Nb-, and Pt-precursor on Si/SiO${}_{2}$ as substrate material.

Precursor	$E_{A}$ (eV)	$\kappa_{0}$ (s${}^{-1}$)
HCo${}_{3}$Fe(CO)${}_{12}$	0.50	$2.9\times 10^{13}$
Nb(NMe${}_{3}$)${}_{2}$(N-*t*-Bu)	0.55	$5.1\times 10^{14}$
Me${}_{3}$CpMePt	0.47	$7.8\times 10^{13}$

## References

[1] Fernández-Pacheco A., Serrano-Ramón L., Michalik J.M., Ibarra M.R., De Teresa J.M., O’Brien L., Petit D., Lee J., Cowburn R.P. (2013). Three dimensional magnetic nanowires grown by focused electron-beam induced deposition. Sci. Rep..

[2] Fernández-Pacheco A., Streubel R., Fruchart O., Hertel R., Fischer P., Cowburn R.P. (2017). Three-dimensional nanomagnetism. Nat. Commun..

[3] Keller L., Al Mamoori M.K.I., Pieper J., Gspan C., Stockem I., Schröder C., Barth S., Winkler R., Plank H., Pohlit M. (2018). Direct-write of free-form building blocks for artificial magnetic 3D lattices. Sci. Rep..

[4] Al Mamoori M., Keller L., Pieper J., Barth S., Winkler R., Plank H., Müller J., Huth M. (2018). Magnetic Characterization of Direct-Write Free-Form Building Blocks for Artificial Magnetic 3D Lattices. Materials.

[5] Esposito M., Tasco V., Cuscunà M., Todisco F., Benedetti A., Tarantini I., Giorgi M.D., Sanvitto D., Passaseo A. (2015). Nanoscale 3D Chiral Plasmonic Helices with Circular Dichroism at Visible Frequencies. ACS Photon..

[6] Winkler R., Schmidt F.P., Haselmann U., Fowlkes J.D., Lewis B.B., Kothleitner G., Rack P.D., Plank H. (2017). Direct-Write 3D Nanoprinting of Plasmonic Structures. ACS Appl. Mater. Interfaces.

[7] Porrati F., Barth S., Sachser R., Dobrovolskiy O.V., Seybert A., Frangakis A.S., Huth M. (2019). Crystalline Niobium Carbide Superconducting Nanowires Prepared by Focused Ion Beam Direct Writing. ACS Nano.

[8] Córdoba R., Mailly D., Rezaev R.O., Smirnova E.I., Schmidt O.G., Fomin V.M., Zeitler U., Guillamón I., Suderow H., De Teresa J.M. (2019). Three-Dimensional Superconducting Nanohelices Grown by He+-Focused-Ion-Beam Direct Writing. Nano Lett..

[9] Huth M., Porrati F., Dobrovolskiy O. (2018). Focused electron beam induced deposition meets materials science. Microelectron. Eng..

[10] Skoric L., Sanz-Hernández D., Meng F., Donnelly C., Merino-Aceituno S., Fernández-Pacheco A. (2019). Layer-by-layer growth of complex-shaped three-dimensional nanostructures with focused electron beams. arXiv.

[11] Fowlkes J.D., Winkler R., Lewis B.B., Stanford M.G., Plank H., Rack P.D. (2016). Simulation-Guided 3D Nanomanufacturing via Focused Electron Beam Induced Deposition. ACS Nano.

[12] Winkler R., Fowlkes J., Rack P., Plank H. (2019). 3D nanoprinting via focused electron beams. J. Appl. Phys..

[13] Córdoba R., Sharma N., Kölling S., Koenraad P.M., Koopmans B. (2016). High-purity 3D nano-objects grown by focused-electron-beam induced deposition. Nanotechnology.

[14] Córdoba R., Ibarra A., Mailly D., De Teresa J.M. (2018). Vertical Growth of Superconducting Crystalline Hollow Nanowires by He+ Focused Ion Beam Induced Deposition. Nano Lett..

[15] Toth M., Lobo C., Friedli V., Szkudlarek A., Utke I. (2015). Continuum models of focused electron beam induced processing. Beilstein J. Nanotechnol..

[16] Porrati F., Pohlit M., Müller J., Barth S., Biegger F., Gspan C., Plank H., Huth M. (2015). Direct writing of CoFe alloy nanostructures by focused electron beam induced deposition from a heteronuclear precursor. Nanotechnology.

[17] Huth M., Porrati F., Schwalb C., Winhold M., Sachser R., Dukic M., Adams J., Fantner G. (2012). Focused Electron Beam Induced Deposition: A Perspective. Beilstein J. Nanotechnol..

[18] Utke I., Hoffmann P., Melngailis J. (2008). Gas-Assisted Focused Electron Beam and Ion Beam Processing and Fabrication. J. Vac. Sci. Technol. B Microelectron. Nanometer Struct. Process. Meas. Phenom..

[19] Friedli V., Utke I. (2009). Optimized molecule supply from nozzle-based gas injection systems for focused electron- and ion-beam induced deposition and etching: simulation and experiment. J. Phys. D Appl. Phys..

[20] Cullen J., Bahm A., Lobo C.J., Ford M.J., Toth M. (2015). Localized Probing of Gas Molecule Adsorption Energies and Desorption Attempt Frequencies. J. Phys. Chem. C.

[21] Engmann S., Stano M., Matejcik S., Ingólfsson O. (2012). Gas phase low energy electron induced decomposition of the focused electron beam induced deposition (FEBID) precursor trimethyl (methylcyclopentadienyl) platinum(iv) (MeCpPtMe3). Phys. Chem. Chem. Phys..

[22] Ragesh Kumar R.K., Bjornsson R., Barth S., Ingólfsson O. (2017). Formation and decay of negative ion states up to 11 eV above the ionization energy of the nanofabrication precursor HFeCo_3_(CO)_12_. Chem. Sci..

[23] Thorman R.M., Ragesh Kumar T.P., Fairbrother D.H., Ingólfsson O. (2015). The role of low-energy electrons in focused electron beam induced deposition: four case studies of representative precursors. Beilstein J. Nanotechnol..

[24] Cullen J., Lobo C.J., Ford M.J., Toth M. (2015). Electron-Beam-Induced Deposition as a Technique for Analysis of Precursor Molecule Diffusion Barriers and Prefactors. ACS Appl. Mater. Interfaces.

[25] Mutunga E., Winkler R., Sattelkow J., Rack P.D., Plank H., Fowlkes J.D. (2019). Impact of Electron-Beam Heating during 3D Nanoprinting. ACS Nano.

[26] Chini P., Colli L., Peraldo M. (1960). Preparazione e proprieta’ dell’idrocarbonile HFeCo_3_(CO)_12_ e di alcuni composti derivati dall’Anione [FeCo_3_(CO)_12_]. Gazz. Chim. Ital..

[27] Baunemann A., Bekermann D., Thiede T.B., Parala H., Winter M., Gemel C., Fischer R.A. (2008). Mixed Amido/Imido/Guanidinato Complexes of Niobium: Potential Precursors for MOCVD of Niobium Nitride Thin Films. Dalton Trans..

